# Current Diagnostic and Treatment Methods of Alzheimer’s Disease: A Narrative Review

**DOI:** 10.7759/cureus.45649

**Published:** 2023-09-20

**Authors:** Rajshri Dafre, Praful Wasnik

**Affiliations:** 1 Health Science, Jawaharlal Nehru Medical College, Wardha, IND; 2 Medicine, Jawaharlal Nehru Medical College, Wardha, IND

**Keywords:** detection of a.d, risk factors, tangles, plaques, alzheimer’s

## Abstract

To diagnose and predict the possibility of Alzheimer's or a different kind of dementia, medical professionals employ tests that look at a patient's mental competence; however, such methods are impracticable. A reliable diagnosis at the start of treatment is essential for therapy. Except in situations with apparent genetic variations, most Alzheimer's patients lack a known etiology. Therefore, every Alzheimer's patient receives the same treatment plan, regardless of the etiology, which may or may not be successful in slowing or preventing the disease's progression. Tau pathology is further complicated by the amyloid buildup that arises from the cellular phase of Alzheimer's disease (AD). Alzheimer's is a degenerative, diverse, complicated, and incurable neurological disorder primarily affecting elderly individuals. The currently accepted drugs available for treating AD, which involve cholinesterase inhibitors and N-methyl-D-aspartate* (*NMDA)-receptor antagonists only provide temporary relief from symptoms. The neurological disorder primarily affecting elderly individuals is degenerative, diverse, complicated, and incurable. Accurate diagnosis is the most essential prerequisite before beginning therapy. Most Alzheimer's patients' causes are still unclear, except for instances where hereditary variations have been noted. The gut microbiota composition significantly influences AD and any age-associated neurological illness. Therapies are very useful in improving the cognitive functions of AD. New microbiota-based therapy alternatives may now be available due to the more recent connection between the altered gut microbiome and neurodegeneration through the gut microbiota-brain axis.

## Introduction and background

Alzheimer's disease (AD) is one of the leading causes of dementia, but dementia is not one of the leading causes of AD [1]. The protein that creates the 'plaques' and 'tangles' in the brain is termed. Indicators of AD can be seen under a microscope as neurotic plaques generating amyloid beta peptide (A-42) and neurofibrillary tangles (NFTs) made of hyperphosphorylated tau [2]. Out of the more than 40 genetic variables for AD that have already been found, apolipoprotein E (APOE) alleles have the strongest correlation with the illness. Sixty to 80 percent of people are suffering from AD due to the heredity factor [3]. Age is the most significant risk factor, including genetic and environmental factors. Therefore, older people are more likely to experience it. Around 40 million individuals worldwide are affected by one of the most common neurodegenerative illnesses, and it is predicted that by 2050, over 100 million individuals will have AD [4]. AD is a debilitating and degenerative brain condition that costs governments, families, and people a lot of money [5]. Combination therapy, N-methyl-D-aspartate (NMDA)-receptor antagonists, and cholinesterase inhibitors are the approved AD treatments for symptomatic relief. However, other factors, such as neurological inflammation, oxygen deprivation, low blood sugar, vascular dysfunction, approval of aberrant proteins, wrong protein folding, support of aberrant proteins, metal dyshomeostasis, etc., all contribute to the pathogenesis of AD. Therefore, these new processes could be viable targets for treatments for AD [6]. The development of new disease-modifying medication (DMF) techniques that target extracellular amyloid plaques and intracellular neurofibrillary tangles in addition to other diverse mechanisms is underway. This review article examines the currently available symptomatic treatments and those in development targeting amyloids, tau proteins, neurological inflammation, and mitochondria dysfunction [7]. For patients with AD in any stage, cholinesterase inhibitors are an option, while memantine is an option for those having moderate-to-severe AD. These drugs have been shown to enhance the overall patient and caretaker quality of life when used appropriately throughout the illness, but they do not affect the course of the disease or the rate of decline [8]. The number of people with mild cognitive problems or any form of dementia is steadily increasing. There is an urgent need to implement alternative, non-pharmacological therapies, given that there is no recently effective treatment for dementia and that drug use can have various adverse effects. This study's objective is to learn people's about how physical activity affects older people's cognitive decline and how it can detect and diagnose AD. Furthermore, an asymmetry of the gut microbiota is thought to play a role in the etiology of several gastrointestinal and extraintestinal illnesses. New microbiota-based therapy alternatives may now be available due to the more recent connection between the altered gut microbiome and neurodegeneration through the gut microbiota-brain axis [9,10].

## Review

Search methodology

**Figure 1:**
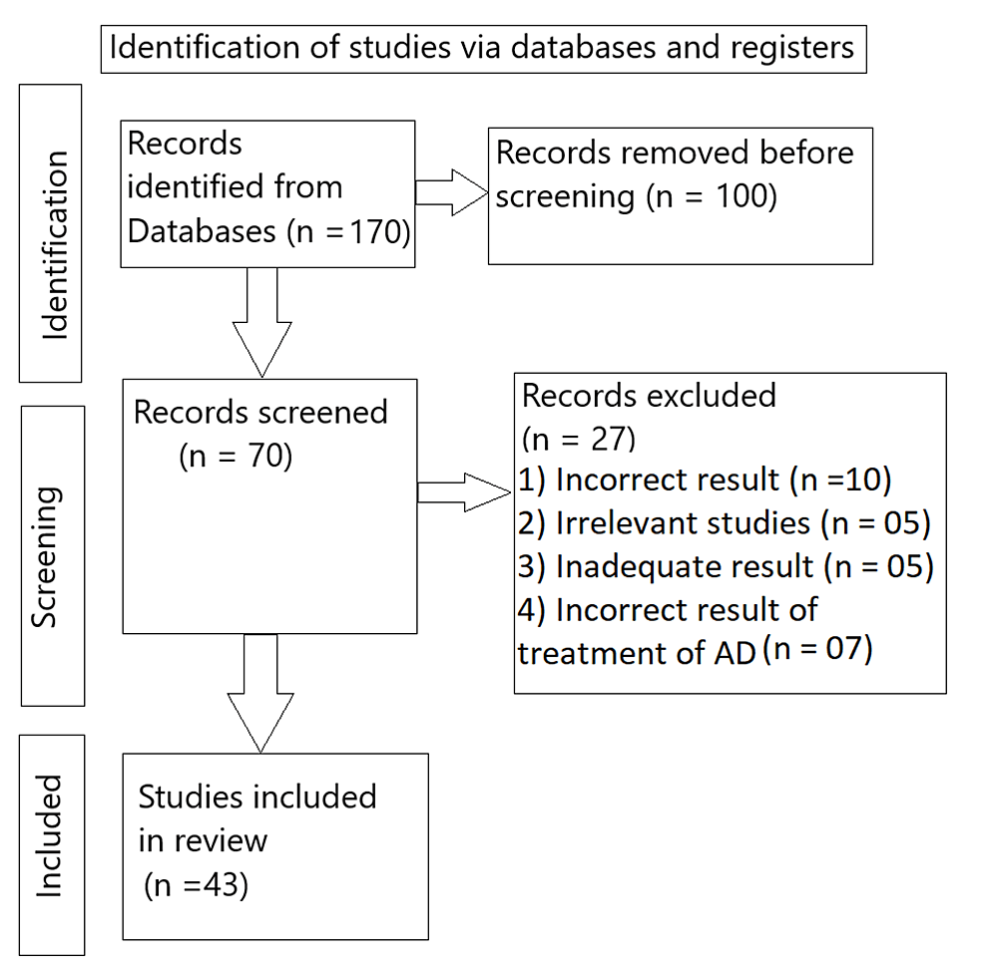
PRISMA flow diagram illustrating the process of study selection of Alzheimer's disease.

We undertook a systematic search through PubMed And CENTRAL in July 2023 using keywords such as "Alzheimer's", "risk factors", and "diagnosis of AD". [Alzheimer's (Title/Abstract)] or [Alzheimer’s(Title /Abstract)] or [Alzheimer’s (MeSH TERMS)]. We additionally searched for key references from bibliographies of the relevant studies. One reviewer independently monitored the retrieved studies against the inclusion criteria, in the beginning, based on the title and abstract and then on full texts. The selection of studies (Figure 1) depended on the following inclusion criteria: (1) patient with AD; (2) recent treatment strategies for AD; (3) new methods for the diagnosis of AD; (4) English language; (5) systematic reviews (6) comprehensive review (7) original article. The following were the exclusion criteria: (1) case study; (2) non-English language articles; (3) opinion articles; and (4) surveys.

Stages of AD

The pre-clinical or pre-symptomatic stage of AD, which can extend for years or longer, is the first of the illness's clinical phases. Mild memory loss, early degenerative alterations in the brain and hippocampus, no functional impairment in daily activities, and no clinical signs or symptoms of AD are characteristics of this stage. 

Mild AD

The illness affects the lateral temporal and parietal lobes when AD is moderate. In this stage, the primary symptoms included reading challenges, poor object recognition, and impaired direction sense appeared [11].

Moderate AD

Frontal lobes are impacted by mild AD. The patient exhibits poor judgment, impulsivity, and lack of focus at this stage [12].

Severe AD

In severe AD, the disease spreads to the brain's occipital lobe. The main symptom seen in this disease is visual problems [13].

Predisposing factors of AD

Risk factors for AD are age, genetics, epigenetics, metals, traumatic brain damage, variation in nutrition, psychiatric variables, infectious agents, vascular illness, immune system, and mitochondrial dysfunction [14].

Age 

In AD most dangerous risk factor is the age for cognitive impairment. The prevalence chances of AD increase with increasing age, estimated to be 19% in persons between the ages of 75 and 84 and 30 to 35%, or even up to 50%, in those over the age of 85 [15].

*Genes * 

While there were difficulties in comprehending early studies on family cases, such as predisposed family memories and awareness of the underlying illnesses implicated, they were a convincing indication that close relatives of individuals with AD had an increased risk of dementia themselves. Heyman et al. emphasized in a prior study that each person's genetic relationship's strength was unique [14].

Metals 

Additionally, a significant portion of the population regularly comes into contact with trace metals, including copper, lead, iron, and aluminum, which can all be risk factors for AD. These metals come from a variety of sources [16].

Traumatic Brain Injury 

Amyloid precursor protein (APP) is seen in cells of neurons and dystrophic neurites around A deposits in head injury survivors, as seen in AD. The discovery of chronic traumatic encephalopathy (CTE), a type of dementia thought to be caused directly by recurrent brain injury, has brought attention to the link between traumatic brain injury (TBI) and chronic traumatic encephalopathy (CTE ) is a tauopathy [17].

*Diet* 

Many of these risk variables for AD have been discovered in recent studies, including an elevated risk with obesity and diet variability. The researchers claim that one AD risk factor could be starvation [18].

*Obesity * 

Obesity is one of the risk factors for AD. Depressive disorders, being overweight, and elevated cholesterol levels, compared to the remaining fourteen risk factors, were more likely to correspond with reduced AD risk, with drug and alcohol consumption being the only one to increase the chance of cognitive decline in an extensive number of cases [19].

*Diabetes*****

It is not unexpected that diabetes has been related to AD because insulin plays a crucial function as a neuromodulator [20].

Mitochondrial Dysfunction** **

Henderson does not address this issue particularly, although there is a long history of speculations linking mitochondrial malfunction to Alzheimer's. Consequently, early AD is characterized by deformed mitochondria and reduced brain metabolic rate [14].

Infectious Agent 

One of the earliest studies to hypothesize that an infection might increase the chance of AD suggested that neurofibrillary tangle (NFT) may be caused by the herpes simplex virus (HSV), an invader to which antibodies may be seen in the cerebrospinal fluid (CSF) in AD. However, more recent studies have examined the DNA of AD patients' brains and peripheral blood leucocytes for human Epstein-Barr virus (EBS), cytomegalovirus (CMV), and human herpes virus 6 (HHV-6), with the results indicating that HHV-6 and Human herpesvirus (EPV) may be risk factors for the development of AD and in cognitive impairment [21].

Psychiatric Factors 

The coexistence of the two diseases may be due to changed levels of common metabolites in the blood, including serum 25-hydroxyvitamin D, tumor necrosis factor (TNF), and cytokine K6. In early life, Increasing stress has been identified as a risk factor for memory loss in the agedness because there is evidence that stress may cause amyloid formation in early anxiety in animals [22].

Methods for the detection of Alzheimer's disease

CSF Proteins (Cerebrospinal Fluid) 

The area of cerebrospinal fluid biomarkers has seen the introduction of innovative phosphorylated tau (p-tau) tests that more accurately represent tau tangle burden than existing cerebrospinal fluid biomarkers of tau disease [23,24]. In 1990, It was observed that a combined analysis of Aβ42 and Aβ40 improved the diagnostic accuracy of AD [25]. After this, several studies have demonstrated that, although cerebrospinal fluid Aβ40 exhibits little to no alteration in Alzheimer's disease, the cerebrospinal fluid Aβ42/Aβ40 ratio performs better as an AD biomarker than cerebrospinal fluid Aβ42 alone. According to recent research, the cerebrospinal fluid Aβ42/Aβ40 ratio has prognostic relevance in the clinical environment and more significant agreement with amyloid positron emission tomography positive [26].

*Blood and Urine Biomarkers of AD* 

Scientists have discovered that levels of two proteins called Aβ42 and Aβ40 in the blood (plasma) can tell us something important about AD [27]. Plasma levels of A-42 and A-40 are now recognised indicators for AD. In AD transgenic mouse models, levels of A42 and A40 in the plasma and CSF rise with age but fall with the accumulation of A in the brain, which triggers the beginning of cognitive impairment. The predictive relevance of A levels in senior individuals was established by Schupf et al. (2008), who also looked into how these biomarkers changed over time with the onset of cognitive impairment or AD. Amide I is a biomarker that can identify AD in people even before they have clinical symptoms of dementia. The test was positive with a likelihood ratio of 7.9 (ESTHER: Alzheimer's Society in Toronto), indicating strong support for this biomarker's ability to detect AD in the general population [2].

Lipid Biomarkers of AD Present in the Blood 

In the central nervous system, the brain lipids are an essential molecule that plays various physiological and biological roles in impulsion conduction and cell signaling. Some brain lipids are phosphatidylcholine, glucosyl ceramides, cholesterol, phosphatidylcholine, and sulfatides. The central nervous system astrocytes, including oligodendrocytes and astrocytes, microglial cells, and neurons, all contain these brain lipids. Additionally, found in myelin, they promote proper conduction, which aids in nerve inflow. The lipid mentioned above indicators may be identified and investigated as a less intrusive diagnostic technique and a means of predicting future illness development and treatment response in AD [28,29].

Brain-Derived Neurotrophic Factor 

Research on memory deterioration in healthy persons is currently associated with high Increasedβ levels and decreased hippocampus volume (HV); however, in the preliminary stages of AD, brain-derived neurotrophic factor (BDNF), exacerbates these circumstances more [30]. In this study, researchers looked at healthy adults with high protein levels called Aβ. First, they found that those adults with both high Aβ levels and the met allele showed a more significant reduction in something called hippocampal volume (HV). HV is a fancy way of talking about a specific part of our brain called the hippocampus. It's like the memory center of our brain. This finding is essential because it gives us clues about what might happen in the early stages of AD.

*Kidney/Brain (KIBRA) Protein, a Memory-Associated Protein* 

Transgenic mice that can acetylate K274 and K281 exhibit memory deficits and impaired long-term hippocampal potential (LTP). LTP deficiency can be rescued by increased actin polymerization or by KIBRA protein expression. Increased tau acetylation is associated with loss of KIBRA in AD. This research has shown a brand-new way that pathogenic tau contributes to cognitive decline and synaptic dysfunction in AD. [31]. 

*Brain Imaging* 

In aging, either spontaneously or with AD, the brain experiences significant structural and functional changes. In AD, it was observed that there was a loss of cortical neurons, with a loss of connectivity in the CNS system. Recent advances in brain imaging have supported unique disruptions in functional neural networks. With the help of brain imaging techniques, identify the brain's functional, molecular, and structural aspects in AD [32].

Recent methods for the treatment of AD

While the specific cause of AD is unknown, treatments that decrease AD risk are available. The patient suffering from AD pharmacological treatment is available. Galantamine, donepezil, rivastigmine, and cholinesterase inhibitors are among the medications that are suggested for people with mild, moderate, or severe AD [33]. One of the new techniques involved in detecting AD is gut microbiota and gut microbiota brain axis (GMBA) [34]. Further prospective population-based studies found that older adults who took non-steroidal anti-inflammatory drugs (NSAIDs) had a 71% decreased chance of dying from AD [35].

*Food and Drug Administration* (*FDA) Approved Medicines*

Food and Drug Administration (FDA) Approved Drugs are currently medications for AD that target glutamatergic or cholinergic neurotransmission. These medications merely treat the symptoms. Despite several continuing clinical trials, no cure has a curative impact. These recently created compounds aim to bind to the tau and amyloid proteins [36].

Gene Therapy in AD

Gene therapy treatments aim to address the underlying cause of an illness, typically a defective gene, protein, or piece of DNA, and then enable the cells to heal themselves. It opens up a world of possibilities for gene therapy, which uses viruses to introduce fresh DNA into living cells after identifying many genes implicated in the pathophysiology of AD [37]. In a different study, researchers used a modified virus to introduce the gene Peroxisome proliferator-activated receptor gamma coactivator 1-alpha (PGC1-alpha) into the brains of mice, which slowed the progression of Alzheimer's [38]. The planning of the current study was based on previous research about PGC-1α, which indicated that the same group could reduce the amount of amyloid-β (Aβ) produced in cell culture. The outcomes of a multicenter randomized clinical study include intracerebral gene transfer in individuals with AD [39].

Immunotherapy For AD

According to the pathogenesis of AD, immunotherapy primarily targets amyloid beta plaques. Some immunotherapy approaches are being researched for Alzheimer's, the two main ones are active, and another is passive vaccination [40].

Probiotics And Prebiotics in AD

Many studies are carried out on humans and animals to determine the impact of probiotics and prebiotics on AD. Prebiotics offer the right conditions and nutrients for the growth of gut microorganisms, and probiotics aid in their development [41]. Probiotic supplements should be moderate and not overly intense since they might upset the gut flora, promoting neuroinflammation and immune system activation [42].

The GMBA in AD

It is essential to understand whether GMBA and gut microbiota work in AD. The gut microbiota composition significantly influences AD and any age-associated neurological illness [43]. Aging older people significantly affects the gut microbiota's appearance, encouraging pro-inflammatory bacteria growth [44]. According to recent studies, changes in the gut microbiota directly affect memory loss and actively contribute to the initiation and development of Alzheimer's disease. The GMBA proposed as a potential target for treating illnesses affecting the central nervous system, such as AD has received much attention in medical investigations [45].

Fecal Microbiota Transplantation

Through fecal microbiota transplantation (FMT), a donor's fecal material solution is sent into the recipient's intestinal tract through a nasogastric tube, colonoscopy, or oral medication to modify the recipient's gut microbiota composition. While the encouraging outcomes in mice clearly show that the gut microbiota plays a part in the onset and progression of neurological diseases, further human study is necessary before FMT is suggested as an adjuvant therapy for AD [46].

The beneficial effects of music therapy and reminiscence therapy on people with AD's cognitive abilities

Music therapy is beneficial in improving the cognitive functions of AD. This supports the growing body of studies that show music therapy to be an effective strategy for enhancing executive functioning, attention, and memory in patients with AD dementia. With the help of music therapy, cognitive functions improve, which can enhance the standard lifestyle for patients and their caregivers. [47]. Applying music to satisfy a person's psychological, mental, physical, and social demands is known as music therapy. You can also use active techniques, such as singing, dancing, or applause, and receptive techniques, where the person listens to music to comprehend its emotional content [48].

The benefits of reminiscence therapy vary significantly between contexts and therapeutic modalities and are typically subtle and variable. In dementia patients, Reminiscence therapy (RT) positively affects Quality of Life, thinking, communication, and mood. Studies on residential care facilities show a broad spectrum of benefits, such as enhanced cognition, communication, and a better quality of life. It is constantly demonstrated to help senior citizens who experience depression. Both medicine and other psychological interventions have similar effects. In addition to reducing depression in older individuals, life review may also improve the quality of life and life satisfaction [49].

The limitations of the review include a lack of discussion on counseling and other management. The keywords used couldn’t find the articles. Although many studies have examined the complications of AD, the effects of AD, and the pathophysiology of AD, there is a lack of research on current detection and treatment methods of AD.

Table 1 discusses the short summary or characteristics of articles included in the review.

**Table 1:** A short overview of the articles included in the review AD: Alzheimer's disease, MCI: mild cognitive impairment, KLSE: Kullback - Leibler Divergence Similarity Estimation, DMA: Dimethylarsinic acid, InAs: Inorganic arsenic, KT: Ketogenic diet, NSAIDs: Non-steroidal anti-inflammatory drugs

Author	Year	Country	Type of Article	Findings
Khan et al. [2]	2020	India	Review	In this article, it is discussed how artificial intelligence, theranostics, and involved biomarkers will all have a significant impact on how AD is managed in the future.
Lanfranco et al. [3]	2020	USA	Review	The treatment model outlined here suggests that an intriguing strategy to treat or prevent AD would be to increase lipidation while concurrently lowering lipid-free Apolipoprotein E protein ( apoE) through a combination therapy.
Gupta et al. [4]	2020	India	Research Support	A lot of research is being done to develop novel pharmacological molecules that have the potential to be both an anti-diabetic medicine and a treatment for type 2 diabetes-related Alzheimer's.
Athar et al. [5]	2021	Oman	Review	Cholinesterase inhibitors, NMDA-receptor antagonists, and their combination therapy, which makes up the currently approved medication, only temporarily relieve symptoms. Researchers from all over the world have made sincere attempts to find new targets, learn about, and create novel therapeutic molecules to relieve the symptoms of AD.
Husain et al. [6]	2021	Oman	Review	In this article discusses molecular hybridization techniques used in the last ten years to create anti-AD medications that can hit various targets.
Thiratmatrakul et al. [7]	2014	Thailand	Research Support	As multifunctional anti-Alzheimer's medications, three novel tacrine-carbazole hybrids were created and described.
Weller et al. [8]	2018	USA	Review	Describe recent developments in our knowledge of the clinical diagnosis and management of Alzheimer's disease and provide information on ongoing clinical trials.
Kouloutbani et al. [9]	2019	Greek	Review	Understanding the exact variables that give exercise its therapeutic potential is crucial for the creation of exercise regimens tailored specifically to the treatment of dementia.
Wang et al. [10]	2020	China	Research Support	The KLSE (Kullback-Leibler Divergence Similarity Estimation) indicator recognizes aberrant brain networks that predict a person's probability of developing AD from mild cognitive impairment, possibly making it a therapeutically useful imaging biomarker.
Wattmo et al. [11]	2016	Sweden	Observational Study	This study brought to light the therapeutic significance of instrumental activities of daily living assessments in patients with mild AD and the significance of maximizing clinical practice of cholinesterase inhibitor (ChEI) dosage even in those with moderate AD.
De-Paula et al. [13]	2012	Brazil	Review	In those with mild, pre-dementia symptoms, new technologies based on structural and functional neuroimaging, as well as the biochemical examination of Cerebrospinal fluid fluid, may be able to show correlations of intracerebral amyloidosis. These techniques, which are also known as AD-related biomarkers, provide strong diagnostic accuracy for identifying people who are at high risk of developing AD.
Henderson et al. [14]	1988	Canberra	Review	This review looks at the supporting evidence for them. An effort is made to pinpoint a common mechanism by which those that seem to be real risk factors could really contribute.
Ferri et al. [15]	2005	USA	Research Support	Shows the breadth of the data from global research on the incidence of dementia.
Armstrong et al. [16]	2013	UK	Review	According to the theory of "allostatic load," AD is a complex condition in which internal and environmental variables interact to speed up the natural aging process.
Gentleman et al. [17]	1993	UK	Research Support	To evaluate if it colocalizes with amyloid protein (βAP) under these circumstances, the immunohistochemistry distribution of the βAP precursor protein (βAPP) was studied in this work.
Tolppanen et al. [19]	2014	Finland	Research Support	analyze the Independent of obesity-related risk factors and co-morbidities, higher midlife body mass index (BMI) is linked to a higher risk of dementia and AD. Higher risk of dementia and AD is linked to a faster decline in BMI and low BMI in late age.
Biessels et al. [20]	2005	Netherlands	Review	The evidence for each of these underlying mechanisms and the link between DM2 and dementia will be examined, with an emphasis on the function of insulin.
Carbone et al. [21]	2014	Italy	Non-U.S. Gov't	Results indicate Epstein–Barr virus and human herpes virus 6 as potential environmental risk factors for cognitive decline and the development of AD in elderly people.
Armstrong et al. [22]	2019	United Kingdom	Review	In a recent assessment, more than 60 environmental risk factors for AD were found and categorized into six groups: heavy metals, other metals, trace elements, occupational exposure, and miscellaneous. It is still very difficult to understand how so many seemingly unrelated risk factors may cause AD..
Yang et al. [23]	2018	Taiwan	Research Support	The propensity-score-matching method identified the substantial correlations between high InAs% or low dimethylarsinic acid (DMA%) and increased AD risk.
Ossenkoppele et al. [24]	2022	Netherlands	Review	Tau aggregate deposition is a pathogenic feature of Alzheimer's disease that is spatially and temporally connected to the onset of neurodegeneration and the appearance of clinical symptoms.
Ashton et al. [25]	2018	UK	Review	They are two well-known Aβ biomarkers that are frequently used: Positron emission tomography A-binding ligands and immunoassays to quantify Aβ42 in cerebrospinal fluid
Schupf et al. [26]	2008	USA	Research Support	A higher plasma Aβ42 level at the start of the investigation was linked to a tripled risk of AD.
Otaegui-Arrazola et al. [27]	2010	Spain	Review	There is still more study to be done in the domain of oxysterols. While there is a dearth of scientific information about the potential toxicity of phytosterol oxidation products, the connection between Cholesterol oxidation products and a number of harmful outcomes is evident.
Zarrouk et al. [28]	2018	France	Review	There is growing evidence that lipids play key roles in the etiology of AD and that certain of them may be used to predict outcomes and make diagnoses.
Lim et al. [29]	2013	Australia	Research Support	Associations between brain-derived neurotrophic factor and AD may be more pronounced in preclinical phases, when the illness manifests nearly solely as minor memory problems, due to the intricacy of AD pathological alterations increasing with disease severity.
Johnson et al. [31]	2012	USA		Over the last four decades, imaging has played a number of roles in the research of Alzheimer disease (AD). To rule out further dementia causes, computed tomography (CT) and later magnetic resonance imaging (MRI) were initially utilized as diagnostic tools.
Breijyeh et al. [32]	2020	Palestine	Review	Early administration of AD medication and patient monitoring for disease progression via biomarker diagnosis are essential for treatment effectiveness. The development of AD pathology may be slowed by future treatments that target tau pathology and the use of combination therapy.
Grossberg et al. [33]	2013	USA	Multicenter Study	Extended-release memantine was efficacious, safe, and well tolerated in this population
Varesi et al. [34]	2022	Italy	Review	In addition, imbalance of the gut microbiota is thought to have a role in the etiology of a number of gastrointestinal and extraintestinal illnesses.
Guzior et al. [35]	2015	Poland	Review	Most novel compounds have heterodimeric structures that enable them to interact with many targets by combining several pharmacophores, whether they are wholly new or derived from natural products or currently used medications (tacrine, donepezil, galantamine, memantine).
Benito-León et al. [36]	2019	Spain	Research Support	The goal was to ascertain whether using NSAIDs was linked to a lower risk of AD mortality.
Tuszynski et al. [37]	2015	California	Clinical Trial	These results suggest that neurons in the aging brain still have the capacity to respond to growth stimuli by sprouting axons, growing cells, and activating functional markers.
Katsouri et al. [38]	2016	United Kingdom	Research Support	The decrease in A pathology and neuroinflammation was the primary reason of the neuroprotective benefits, since wild-type animals given the identical medication showed no adverse consequences.
Rafii et al. [39]	2018	San Diego	Review	This multicenter randomized clinical experiment proved the viability of stereotactic gene transfer research in AD patients under sham surgery control. Although well tolerated, administration had no impact on chosen AD biomarkers or clinical outcomes. It is necessary to get pathological evidence of precise gene targeting.
Schenk et al. [40]	2002	USA	Review	The current treatments for Alzheimer's disease only address the signs of neurodegeneration and, at most, produce modest, transient improvements in cognitive performance.
Naomi et al. [41]	2021	Malaysia	Review	According to this claim, there have been no negative effects associated with the use of probiotics for AD. Therefore, further clinical studies must be undertaken in the future to find AD-specific alterations in the gut microbiota that might reveal fresh information about probiotics as a promising therapeutic target.
Leblhuber et al. [42]	2018	Germany	Review	According to the findings, taking a multispecies probiotic supplement with Alzheimer's disease patients alters the makeup of their gut bacteria and how tryptophan is metabolized in their blood.
Lin et al. [43]	2017	China	Review	Recently, the idea of Multi-Target-Directed Ligands (MTDLs) has developed as a novel method for creating therapeutic agents for AD, taking into account the disease's multifactorial character.
Cryan et al. [44]	2019	Ireland	Review	In domains looking into the molecular and physiological causes of psychiatric, neurodevelopmental, aging-related, and neurodegenerative illnesses, this axis is gaining ground.
Gupta et al. [45]	2016	Canada	Review	The infusion of a solution of fecal matter from a donor into the intestinal tract of a recipient is known as fecal microbiota transplantation (FMT), which aims to directly alter the recipient's gut microbial composition and provide health benefits.
Ma et al. [46]	2018	USA	Research Support	Cognitive performance is significantly influenced by neurovascular integrity, including cerebral blood flow (CBF) and blood-brain barrier (BBB) function.
Bleibel et al. [47]	2023	France	Review	The findings of this analysis indicate the potential advantages of music therapy as an additional treatment choice for people with AD and the significance of further research in this area.

## Conclusions

Millions of people worldwide are affected by AD, a complicated neurological disease. Memory loss and cognitive, linguistic, and problem-solving challenges are all symptoms of this disease. Over the past two decades, many biochemical and pharmacological investigations have been carried out to better understand the complexity of this progressive disease. New methods of detection of AD are available and include brain lipids, brain-derived neurotropic factors, KIBRA, lipids bookmarks present in blood, blood and urine biomarkers, and CSF Fluid. They play a significant role in AD management in the future. Recent treatment methods are available: FDA-approved medicine, gene therapy, probiotics and prebiotics, immunotherapy, GMBA, and fecal microbiota. This article aims to make people aware of treatment strategies and improved quality of life and life satisfaction.
